# Expression Profiling Predicts Survival in Kidney Cancer

**DOI:** 10.1371/journal.pmed.0030035

**Published:** 2005-12-06

Nearly 95,000 people worldwide die from kidney cancer every year, and renal cell carcinoma (RCC) accounts for most of the deaths. Surgery can cure 60%–70% of patients with localized disease and prolong survival in patients with metastatic disease, but survival rates after treatment have not improved appreciably over the past 30 years.

Current survival estimates based on clinical characteristics such as tumor size and grade are not very accurate, and the varied response to surgery and other treatments suggests an underlying diversity that is not captured by the clinical parameters. As has been the case for other cancer types, researchers hope that comprehensive molecular genetic analysis will reveal distinguishing features that could serve as prognostic indicators, improve outcome prediction, and inform treatment decisions. Several previously reported expression-profiling studies of relatively small sets of RCCs suggested that comprehensive molecular genetic analysis might be useful in this cancer type as well.

To further the understanding of the genetics and molecular biology underlying RCC, James Brooks and colleagues determined the gene expression patterns of a set of 177 tumors from patients with detailed clinical information available, including long-term follow-up. Based on the results, the researchers could divide the tumors into five distinct subgroups, which differed in the expression patterns of over 3,000 genes. These subgroups correlated with survival after nephrectomy. The correlation was independent of tumor stage and grade, suggesting that molecular and genetic changes early during tumorigenesis determine the characteristics of a particular cancer and can be used to predict clinical outcome.

Brooks and colleagues then used a computational tool to identify 259 genes for which expression status was highly predictive of clinical outcome. The genes in this prognostic set represent a range of molecular pathways, and map to different parts of the genome. They found that 95% of them are expressed at high levels in tumors of patients with a good prognosis and at low levels in more aggressive cancers.

In an independent group of patients, the researchers used these 259 genes to calculate a risk score, and showed that it predicted patient survival independent of clinical parameters. They suggest that combining this risk score with tumor grade, stage, and patient performance status might help to identify patients with RCC who have a high probability of being cured and need less intensive adjuvant treatment and follow-up testing after surgery, as well as those who should receive more aggressive treatment. [Fig pmed-0030035-g001]

**Figure pmed-0030035-g001:**
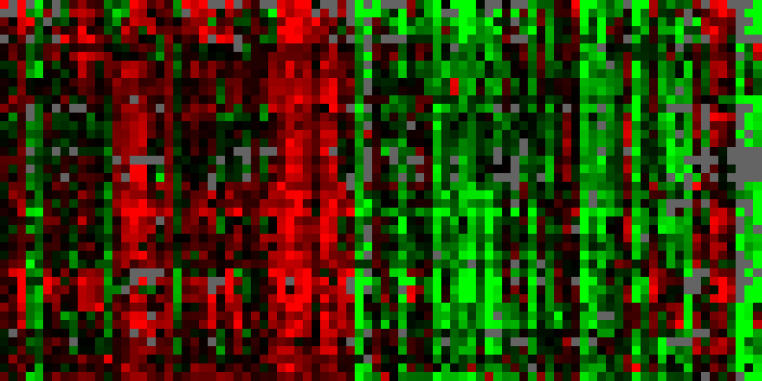
A group of renal cell carcinomas share similar expression patterns

The next step will be to test the value of the risk score in independent studies. To be able to determine expression profiles in routine clinical settings, it will also be necessary to further reduce the number of genes in the prognostic set. Brooks et al. find that as few as four genes from their prognostic set can estimate outcome. They acknowledge that “application of the [supervised principal components] risk score in the clinical setting will depend on independent confirmation of [their] findings,” but conclude “that it should be possible to develop clinically useful predictors of survival based on these technologies.”

